# The effect of education debt on PAs' specialty choice or preference

**DOI:** 10.1097/01.JAA.0000000000000166

**Published:** 2024-12-19

**Authors:** Andrzej Kozikowski, Mirela Bruza-Augatis, Dawn Morton-Rias, Kasey Puckett, Colette Jeffery, Alicia Quella, Sheila Mauldin, Joshua Goodman

**Affiliations:** At the National Commission on Certification of Physician Assistants (NCCPA), **Andrzej Kozikowski** is senior director of research, **Mirela Bruza-Augatis** is a research scientist, **Dawn Morton-Rias** is president and chief executive officer, **Kasey Puckett** is a research analyst, **Colette Jeffery** is a senior research analyst, **Alicia Quella** is director of communications and PA relations, **Sheila Mauldin** is a senior assessment advisor, and **Joshua Goodman** is vice president of research and exam programs. The authors have disclosed no potential conflicts of interest, financial or otherwise.

**Keywords:** education debt, physician associate/assistant, medical specialty, nonprimary care, primary care, PA students

## Abstract

**Objective:**

This study investigated educational debt, repayment strategies, and other factors potentially related to recently certified physician associates/assistants' (PAs') choice or preference for primary care versus all other specialties.

**Methods:**

A national dataset from 2023 of recently certified PAs was used to conduct quantitative and qualitative analyses. Analyses were conducted separately for PAs who had accepted a clinical position and those who had not accepted a clinical position.

**Results:**

Overall, 88% of respondents reported having educational debt, with 13.5% owing $200,000 or more. PAs from backgrounds underrepresented in medicine had accrued higher educational debt than their counterparts. Educational debt was significantly associated with specialty choices/preferences: PAs with higher debt were more likely to opt for nonprimary care specialties. Qualitative analyses revealed that PAs rely on different strategies for repaying their loans, depending on whether they choose or prefer primary care or nonprimary care specialties.

**Conclusions:**

These results suggest that educational debt plays a role in specialty selection, but other factors also are significant.

The escalating burden of educational debt among US healthcare professionals is a growing concern. A recent comprehensive review based on 812 studies from 1990 to 2022 underscored that this topic is gaining attention and is increasingly studied, particularly within medicine.^1^ The Association of American Medical Colleges (AAMC) reported that median debt for medical school graduates increased 25%, from $160,000 in 2009 to $200,000 in 2019.^2^ Similar trends of increased educational debt have been observed for physician associate/assistant (PA) students. Data from the PA Education Association (PAEA) revealed a rise in the proportion of PA students anticipating at least $100,000 in debt, from 39% in 2013 to 63.9% in 2021.^3^,^4^ In 2021, PAEA reported that 5.3% of PA students expected to repay education loans of $200,000 or more.^4^ Reports from the National Commission on Certification of Physician Assistants' (NCCPA) Statistical Profile of Recently Board Certified PAs found that in 2013, 51.9% of newly certified PAs had educational debt of more than $100,000.^5^ By 2022, 63.6% of PAs reported starting their careers with at least $100,000 in debt and 12.4% reported debt exceeding $200,000.^6^ These estimates suggest a rapid increase in educational debt burden—a concerning trend with potentially negative repercussions.

One potential effect of increasing medical education debt is its influence on career paths: It may steer some clinicians with substantial debt away from primary care to more lucrative specialized disciplines.^7^,^8^ This, in turn, can further exacerbate the ongoing shortage of primary care providers and reduce healthcare access, particularly for underserved communities.^9^ Another concern is the effect of higher educational debt burdens on clinicians from racial/ethnic groups underrepresented in medicine (URiM) and whether this could lead to further decreasing the diversity of the healthcare workforce.^10^,^11^ Holaday and colleagues studied debt among medical residents by race and ethnicity, including premedical education, medical education, and consumer debt.^11^ The authors found that medical residents from URiM groups were more likely to report medical education debt than those from non-URiM groups. Specifically, medical residents who self-identified as Black had four times higher odds of reporting any type of debt.^11^

Research examining the relationship between educational debt and specialty choice among medical students and residents reveals a potentially complex and nuanced picture. A 2019 systematic review indicated that, for the most part, higher debt levels among medical students were linked with a preference for higher-paying specialties.^12^ However, significant heterogeneity existed among the studies included in the review: 20 showed that higher debt was associated with choosing higher-paying specialties, 8 with primary care and academic medicine, and 9 found no correlation.^12^ A 2023 systematic review by Lin and colleagues found that higher debt may disincentivize medical and surgical residents from pursuing subspecialty training and academic career paths.^13^ The authors also noted substantial variation in the studies included in the review.

Lapidus and colleagues identified a different potential effect of debt.^14^ Their study found that medical students with debt exceeding $300,000, particularly those from Black or Hispanic/Latinx backgrounds, were more likely than their counterparts to consider practicing in underserved areas. This implies that the effect of educational debt on specialty choice is complex and varies depending on the student's background, preferences, financial burden, repayment strategies, and specific circumstances.^15^

Although research on PA educational debt and specialty choice is not as extensive as that with medical students and physicians, it has a substantial foundation spanning a few decades. Early work by Singer and Hooker in 1996 identified income as an important factor influencing specialty selection for PAs in nonprimary care settings; loan repayment and scholarship program availability were determinants for PAs in primary care.^16^ However, intrinsic interest in the activities of a given specialty outweighed financial factors. In 2012, Diemer and colleagues determined that educational debt did not influence PAs' decisions about providing care in rural Texas.^17^ In 2013, a PAEA report examined the student debt of first-year PA students, finding that 88% of respondents indicated that the ability to pay off debt was important, and 72% said that earning potential was important.^3^ In a 2014 study, Snyder and colleagues revealed that although debt may not initially affect specialty choice, a significant portion of urban PAs would have considered rural practice had debt forgiveness programs been available.^18^ Also, in 2014, the Robert Graham Center conducted a mixed-method study commissioned by PAEA that investigated the role of educational debt on PA career decisions.^19^ The study found that clinical rotations had a significant role in shaping the career choices of PA students, and that interactions with preceptors were particularly effective. Interestingly, the research also uncovered that a considerable number of PA students were undecided on their specialty choice, even just weeks away from graduation. The authors stated that the immediate necessity to begin repaying educational loans postgraduation could influence PAs to prioritize their debt when deciding on their specialty.

More recently, in 2019, Twombly and colleagues found that educational debt and income were the primary factors driving the selection of PAs' first positions.^20^ These findings suggest that PAs may employ different strategies for addressing educational debt: some may pursue higher-paying specialties and others may rely on loan repayment options available in primary care. Another critical factor to consider is that PAs can easily change specialties, and thus, are not locked into particular disciplines for their entire careers.^21^,^22^ The PA profession's flexibility in switching specialties may influence career trajectories, allowing clinicians to pay off debt while working in one field before transitioning to another.

These diverse findings highlight educational debt's complex and multifaceted role in specialty choice among PAs. Further research is necessary to understand how student debt intersects with personal characteristics and repayment strategies to shape PAs' specialty choices and preferences. Given the existing gaps in research on this crucial topic, we aimed to examine the effect of educational debt and other pertinent factors related to recently certified PAs' choice or preference for primary care versus all other specialties.

## METHODS

We analyzed quantitative and qualitative data from the NCCPA's Recently Certified PA module of the PA Professional Profile. This module gathers information from newly certified PAs during the first year of their initial certification, providing insights into early career workforce patterns. NCCPA reported that 11,762 PAs were certified for the first time in 2023, and 7,494 (63.7%) of PAs responded to whether they accepted a PA position. We analyzed data from all early-career PAs as well as separately for two subgroups: those who had accepted their first PA position and those who had not (either actively searching or not yet searching).

PAs who had accepted a position (n = 4,538; 60.6%) were asked about the specialty of the new position, if their educational debt influenced their decision, whether an educational loan repayment incentive was offered, and their starting income. PAs who had not accepted a position (n = 2,956; 39.4%) were asked about their preferred specialty, whether their preference was affected by their educational debt, whether they were seeking a position that offered educational loan repayment, and the minimum starting salary they would accept. We also explored potential differences in specialty choice for PAs (primary care versus all other specialties) for both subgroups by demographics (age, sex, race/ethnicity, US region, urban/rural setting) and total (undergraduate and graduate) educational debt.

Two open-ended questions provided us with the opportunity for richer examinations of the perceived influence of educational debt on specialty choice or preference. PAs who responded affirmatively that their educational debt influenced their specialty choice or preference were prompted to describe how it affected their choice. We conducted separate thematic analyses of the open-ended comments from four groups:

- PAs who accepted a position in primary care
- PAs who accepted a position in other specialties
- PAs who had not accepted a position but prefer primary care
- PAs who have not accepted a position but prefer other specialties.

We conducted descriptive statistics (counts, proportions, means, and standard deviations) and bivariate analyses (ANOVA with post hoc Scheffe tests, *t-*tests, and *chi*-square tests, as appropriate). All quantitative analyses were conducted using R statistical software. Qualitative thematic analyses of responses to the open-ended items were conducted in NVivo. Sterling IRB determined that the study was exempt.

## RESULTS

Overall, 12% of recently certified PAs reported no educational debt upon graduation from PA school (Table 1). Debt levels varied, with 24.2% accruing up to $100,000; 25.8% accruing $100,000 to $150,000; 24.5% accruing $150,000 to $199,000; and 13.5% reporting debt exceeding $200,000. We detected significant differences in the amount of educational debt accrued by age (*P* < .001), race (*P* < .001), ethnicity (*P* = .001), US region (*P* < .001), and whether PAs had accepted a position or were still searching (*P* = .001). We followed up the significant ANOVA results of educational debt by age with post hoc Scheffe tests. PAs who accrued $200,000 or more in debt were older (mean age, 29.9 years) than those who accrued $150,000 to $199,999 (mean age, 28 years; *P* < .001), $100,000 to $149,999 (mean age, 27.6 years; *P* < .001), less than $100,000 (mean age, 27.4 years; *P* < .001), or had no educational debt (mean age, 28.3 years; *P* = .023). Table 2 depicts the differences in debt accrued by race (*P* < .001). PAs who self-identified as Asian had the highest proportion (14.2%) of indicating no debt; Black PAs had the lowest (7.1%). For the highest level of educational debt ($200,000 or more), PAs who identified as White (12.7%) and Asian (12.7%) had the lowest proportion; those who identified as other or Black had the highest proportions (23.4% and 20.6%), respectively.

**TABLE 1.** Educational debt of recently certified PAs by demographic characteristics

	None (n = 820; 12%)	Less than $100,000 (n = 1,658; 24.2%)	$100,000 to $149,000 (n = 1,763; 25.8%)	$150,000 to $199,999 (n = 1,677; 24.5%)	$200,000 or more (n = 922; 13.5%)	*P* value
**Age**						
Mean (SD)	28.3 (4.8)	27.4 (4.1)	27.6 (4.1)	28 (3.9)	29 (4.5)	< .001
**Sex**						
Female	618 (11.9%)	1,298 (25%)	1,330 (25.7%)	1,236 (23.8%)	702 (13.5%)	.062
Male	197 (12.4%)	351 (22%)	421 (26.4%)	422 (26.5%)	204 (12.8%)	
**Ethnicity**						
Non-Hispanic/Latinx	749 (12.4%)	1,466 (24.4%)	1,554 (25.8%)	1,468 (24.4%)	781 (13%)	.001
Hispanic/Latinx	58 (8.6%)	157 (23.4%)	167 (24.9%)	171 (25.4%)	119 (17.7%)	
**US region**						
South	335 (13.9%)	623 (25.9%)	571 (23.8%)	596 (24.8%)	277 (11.5%)	< .001
Northeast	187 (10.9%)	415 (24.2%)	457 (26.7%)	387 (22.6%)	268 (15.6%)	
Midwest	154 (10.4%)	369 (24.9%)	464 (31.3%)	343 (23.1%)	154 (10.4%)	
West	143 (11.7%)	249 (20.3%)	269 (21.9%)	347 (28.3%)	219 (17.8%)	
**Setting**						
Urban	773 (12.1%)	1,547 (24.1%)	1,648 (25.7%)	1,563 (24.4%)	879 (13.7%)	.147
Rural/isolated	46 (11.4%)	106 (26.2%)	110 (27.2%)	105 (25.9%)	38 (9.4%)	
**Accepted PA position**						
No	273 (10.5%)	613 (23.7%)	680 (26.3%)	627 (26.3%)	395 (15.3%)	.001
Yes	545 (12.8%)	1,045 (24.6%)	1,083 (25.5%)	1,048 (24.7%)	527 (12.4%)	

**TABLE 2.** Differences in educational debt by race (*P* < .001)

Totals may not equal 100% because of rounding.					
Debt	White	Asian	Black	Multiple races	Other∗
None	12%	14.2%	7.1%	12.6%	9.4%
Less than $100,000	24.7%	26.2%	15.4%	19.6%	22.9%
$100,000 to $149,000	26.4%	25.1%	24.5%	20.7%	23.4%
$150,000 to $199,999	24.1%	21.9%	32.4%	31.9%	20.8%
More than $200,000	12.7%	12.7%	20.6%	15.2%	23.4%

∗ Other includes Native Hawaiian/Pacific Islander, Native American, and Alaska Native.

Hispanic/Latinx PAs versus non-Hispanic/Latinx PAs were less likely to report no educational debt (8.6% versus 12.4%; *P* = .001) and more likely to report debt of $200,000 or more (17.7% versus 13%; *P* = .001; Table 1), respectively. More PAs in the South reported no educational debt, compared with those in the West, who had the highest percentage among those reporting debt of $200,000 or more (*P* < .001). Notably, PAs who had accepted a position versus those who had not were more likely to report no educational debt (12.8% versus 10.5%; *P* = .001) and less likely to have $200,000 or more (12.4% versus 15.3%; *P* = .001; Table 1).

## RECENTLY CERTIFIED PAS WHO ACCEPTED A POSITION

Among PAs who had accepted a position, 22.7% chose primary care, and 77.3% opted for all other disciplines (Table 3). Among this group, PAs who selected primary care versus all other specialties were slightly older (mean age, 28.2 years versus 27.6 years; *P* < .001). We also found a significant association between race and specialty selection (*P* < .001). PAs who self-identified as White were less likely to choose primary care compared with all other specialties (77.2% versus 81.8%); however, PAs who self-identified as Asian (10.4% versus 9.2%) and Black (4.8% versus 2.8%) were more likely to choose primary care. Additionally, PAs who identified as Hispanic/Latinx (11.6% versus 8.4%; *P* = .002) and those living in rural areas (11.4% versus 5.5%; *P* < .001) were more likely to accept positions in primary care versus other disciplines. Lastly, in terms of differences by demographics, we also observed that the West was the only US region with a higher proportion of PAs deciding on primary care compared with all other specialties (*P* < .001).

**TABLE 3.** Characteristics of recently certified PAs by specialty chosen

	Primary care (n = 1,002; 22.7%)	All other specialties (n = 3,420; 77.3%)	*P* value
**Age**			
Mean (SD)	28.2 (4.5)	27.6 (3.9)	< .001
**Sex**			
Female	733 (73.7%)	2539 (75.1%)	.419
Male	261 (26.3%)	843 (24.9%)	
**Race**			
White	742 (77.2%)	2712 (81.8%)	< .001
Asian	100 (10.4%)	305 (9.2%)	
Black	46 (4.8%)	93 (2.8%)	
Multiple races	35 (3.6%)	130 (3.9%)	
Other	38 (4%)	74 (2.2%)	
**Ethnicity**			
Non-Hispanic/Latinx	859 (88.4%)	3,059 (91.6%)	.002
Hispanic/Latinx	113 (11.6%)	279 (8.4%)	
**US region**			
South	344 (34.5%)	1,188 (34.8%)	< .001
Northeast	209 (20.9%)	1,001 (29.3%)	
Midwest	207 (20.7%)	758 (22.2%)	
West	238 (23.8%)	470 (13.8%)	
**Setting**			
Urban	881 (88.6%)	3,225 (94.5%)	< .001
Rural/isolated	113 (11.4%)	189 (5.5%)	
**Educational debt**			
None	179 (18.5%)	363 (11.1%)	< .001
Less than $100,000	273 (28.2%)	769 (23.5%)	
$100,000 to $149,999	219 (22.6%)	861 (26.4%)	
$150,000 to $199,999	209 (21.6%)	837 (25.6%)	
$200,000 or more	89 (9.2%)	437 (13.4%)	
**Education debt influenced practice decision**			
No	790 (79.7%)	2,973 (88.1%)	< .001
Yes	201 (20.3%)	402 (11.9%)	
**Starting income**			
Up to $90,000	102 (10.5%)	337 (10.2%)	< .001
$90,001 to $100,000	187 (19.3%)	381 (11.6%)	
$100,001 to $110,000	286 (29.5%)	851 (25.9%)	
$110,001 to $120,000	181 (18.7%)	727 (22.1%)	
$120,001 to $130,000	109 (11.2%)	560 (17%)	
More than $130,000	105 (10.8%)	435 (13.2%)	
**Incentive for education loan repayment influenced decision to accept position**			
No	790 (78.8%)	3148 (92%)	< .001
Yes	212 (21.2%)	272 (8%)

When evaluating the relationship between the amount of educational debt accrued and the specialty chosen, we found that PAs who accepted a position in primary care reported less debt than those in all other specialties (*P* < .001). For instance, PAs who selected primary care versus their counterparts were more likely to indicate having no educational debt (18.5% versus 11.1%) and less likely to report debt of $200,000 or more (9.2% versus 13.4%). Interestingly, when directly asked whether educational debt influenced their specialty decision, PAs who chose primary care versus other disciplines were more likely to say *yes* (20.3% versus 11.9%; *P* < .001) and also to report that the position they accepted offered loan repayment as an incentive (21.2% versus 8%; *P* < .001). Regarding starting income, PAs who selected primary care versus their peers who chose nonprimary care specialties were more likely to report lower income ranges (*P* < .001).

Table 4 provides a detailed characterization of PA attributes parsed by self-reporting and whether educational debt influenced their specialty choice for their accepted position. About 14% said that debt influenced their specialty decision. In terms of differences by demographics, we observed significant associations with age, race, and US region (all *P* < .001). PAs who were older (mean age 28.5 years versus 27.6 years), identified as Black (5.5% versus 2.9%), identified as other race (5% versus 2.2%), and were from the West US region (22.4% versus 15%) were more likely to indicate that educational debt influenced their decision. Not surprisingly, we found a dose-response pattern of debt accrued and its effect on specialty choice: the likelihood of PAs indicating that debt influenced their decision increased with increasing debt (*P* < .001). Likewise, PAs who said that their educational debt had an effect on their specialty choice were more likely to report higher starting income (*P* = .001) and that their position offered a loan repayment incentive (*P* < .001).

**TABLE 4.** Did your level of educational debt influence your decision to pursue a position in a primary care or specialty practice?

	No (n = 3,766; 86.2%)	Yes (n = 603; 13.8%)	*P* value
**Age**			
Mean (SD)	27.6 (3.9)	28.5 (4.5)	< .001
**Sex**			
Female	2,794 (75%)	438 (73.5%)	.472
Male	933 (25%)	158 (26.5%)	
**Race**			
White	2,975 (81.6%)	444 (76.8%)	< .001
Asian	344 (9.4%)	52 (9%)	
Black	105 (2.9%)	32 (5.5%)	
Multiple races	143 (3.9%)	21 (3.6%)	
Other	81(2.2%)	29 (5%)	
**Ethnicity**			
Non-Hispanic/Latinx	3,342 (90.9%)	532 (90.9%)	1
Hispanic/Latinx	334 (9.1%)	53 (9.1%)	
**US region**			
South	1,300 (34.5%)	207 (34.6%)	< .001
Northeast	1,054 (28%)	148 (24.7%)	
Midwest	846 (22.5%)	110 (18.4%)	
West	563 (15%)	134 (22.4%)	
**Setting**			
Urban	3,503 (93.3%)	548 (91.5%)	.134
Rural/isolated	253 (6.7%)	51 (8.5%)	
**Educational debt**			
None	516 (14.2%)	27 (4.5%)	< .001
Less than $100,000	926 (25.4%)	116 (19.5%)	
$100,000 to $149,999	922 (25.3%)	159 (26.7%)	
$150,000 to $199,999	867 (23.8%)	178 (29.9%)	
$200,000 or more	410 (11.3%)	116 (19.5%)	
**Starting income**			
Up to $90,000	397 (10.8%)	42 (7.1%)	.001
$90,001 to $100,000	494 (13.5%)	73 (12.3%)	
$100,001 to $110,000	991 (27.1%)	144 (24.3%)	
$110,001 to $120,000	777 (21.2%)	129 (21.8%)	
$120,001 to $130,000	563 (15.4%)	104 (17.5%)	
More than $130,000	440 (12%)	101 (17%)	
**Incentive for education loan repayment influenced decision to accept position**			
No	3,419 (90.8%)	469 (77.8%)	< .001
Yes	347 (9.2%)	134 (22.2%)	

## RECENTLY CERTIFIED PAS WHO HAD NOT ACCEPTED A POSITION

Overall, 24.5% of PAs who had not yet accepted a position preferred primary care, and 75.5% favored all other specialties (Table 5). Female PAs (82.2% versus 78%; *P* = .028) and those residing in the West US region (24% versus 19.4%; *P* = .045) were more likely to prefer primary care compared with all other specialties. PAs who had accrued higher educational debt were more likely to prefer nonprimary care specialties (*P* = .037). For example, a more significant proportion of PAs with $200,000 or more in debt preferred nonprimary care specialties versus primary care (16.3% versus 12.4%). When PAs were asked whether educational debt influenced the specialty they would seek, we observed that those preferring other specialties were more likely to respond yes (21.2%) compared with those preferring primary care (15.4%, *P* = .002). PAs who preferred nonprimary care specialties reported that they would accept higher salary ranges for their first clinical PA positions (*P* < .001) but were less likely to look for positions that offered loan repayment as an incentive (41.8% versus 46.9%; *P* = .029).

**TABLE 5.** Characteristics of recently certified PAs by preferred specialty

	Primary care (n = 627; 24.5%)	All other specialties (n = 1,930; 75.5%)	*P* value
**Age**			
Mean (SD)	28.2 (4.7)	28.3 (4.3)	.878
**Sex**			
Female	509 (82.2%)	1,498 (78%)	.028
Male	110 (17.8%)	423 (22%)	
**Race**			
White	423 (70.5%)	1,374 (73.8%)	.34
Asian	87 (14.5%)	268 (14.4%)	
Black	32 (5.3%)	83 (4.5%)	
Multiple races	33 (5.5%)	80 (4.3%)	
Other	25 (4.2%)	57 (3.1%)	
**Ethnicity**			
Non-Hispanic/Latinx	534 (86.8%)	1,680 (89.1%)	.139
Hispanic/Latinx	28 (4.5%)	205 (10.9%)	
**US region**			
South	215 (34.2%)	690 (35.8%)	.045
Northeast	122 (19.5%)	447 (23.2%)	
Midwest	139 (22.2%)	417 (21.6%)	
West	150 (24%)	374 (19.4%)	
**Setting**			
Urban	598 (95.5%)	1,849 (96.1%)	.605
Rural/isolated	28 (4.5%)	75 (3.9%)	
**Educational debt**			
None	63 (10.9%)	183 (10.2%)	.037
Less than $100,000	155 (26.7%)	410 (22.9%)	
$100,000 to $149,999	163 (28.1%)	456 (25.5%)	
$150,000 to $199,999	127 (21.9%)	450 (25.1%)	
$200,000 or more	72 (12.4%)	292 (16.3%)	
**Education debt influenced practice decision**			
No	515 (84.6%)	1,472 (78.8%)	.002
Yes	94 (15.4%)	396 (21.2%)	
**Starting income**			
Up to $90,000	54 (9%)	101 (5.5%)	< .001
$90,001 to $100,000	155 (25.7%)	364 (19.8%)	
$100,001 to $110,000	225 (37.4%)	640 (34.8%)	
$110,001 to $120,000	84 (14%)	362 (19.7%)	
$120,001 to $130,000	49 (8.1%)	244 (13.3%)	
More than $130,000	35 (5.8%)	129 (7%)	
**Incentive for education loan repayment influenced decision to accept position**			
No	333 (53.1%)	1,123 (58.2%)	.029
Yes	294 (46.9%)	807 (41.8%)	

Table 6 shows PA demographics and preferences for positions and how those data were associated with self-reporting on whether educational debt affected the specialty respondents planned to pursue. About 20% acknowledged that the amount of educational debt they had accrued affected which specialty they would pursue for their first position. We found significant differences by age (*P* < .001), sex (*P* = .011), ethnicity (*P* = .018), and US region (*P* = .016). PAs who were older (mean age, 29.3 years versus 28 years), male sex (24.6% versus 19.6%), identified as Hispanic/Latinx (14.1% versus 10.4%), and from the South (37.6% versus 35.2%) or West (25% versus 20.3%) were more likely to indicate that debt affected specialty choice. PAs who said that their level of educational debt affected whether they intend to seek a position in primary care or other specialties had more accrued educational debt (*P* < .001), reported higher salary ranges that they would accept (*P* = .001), and were more likely to seek positions offering loan repayment (*P* < .001).

**TABLE 6.** Will your level of educational debt influence your decision about whether to seek a position in primary care or a specialty practice?

	No (n = 2,163; 79.8%)	Yes (n = 546; 20.2%)	*P* value
**Age**			
Mean (SD)	28 (4.3)	29.3 (5.1)	< .001
**Sex**			
Female	1,726 (80.4%)	410 (75.4%)	.011
Male	420 (19.6%)	134 (24.6%)	
**Race**			
White	1,530 (73.1%)	370 (71%)	.08
Asian	318 (15.2%)	69 (13.2%)	
Black	84 (4%)	34 (6.5%)	
Multiple races	94 (4.5%)	27 (5.2%)	
Other	68 (3.3%)	21 (4%)	
**Ethnicity**			
Non-Hispanic/Latinx	1,898 (89.6%)	456 (85.9%)	.018
Hispanic/Latinx	220 (10.4%)	75 (14.1%)	
**US region**			
South	760 (35.2%)	205 (37.6%)	.016
Northeast	487 (22.6%)	102 (18.7%)	
Midwest	474 (22%)	102 (18.7%)	
West	438 (20.3%)	136 (25%)	
**Setting**			
Urban	2,072 (96.2%)	517 (94.9%)	.2
Rural/isolated	82 (3.8%)	28 (5.1%)	
**Educational debt**			
None	263 (12.9%)	8 (1.5%)	< .001
Less than $100,000	528 (25.9%)	83 (15.4%)	
$100,000 to $149,999	547 (26.8%)	131 (24.3%)	
$150,000 to $199,999	473 (23.2%)	154 (28.6%)	
$200,000 or more	231 (11.3%)	163 (30.2%)	
**Starting income**			
Up to $90,000	142 (6.7%)	27 (5%)	.001
$90,001 to $100,000	478 (22.6%)	87 (16.1%)	
$100,001 to $110,000	737 (34.8%)	191 (35.4%)	
$110,001 to $120,000	389 (18.4%)	106 (19.7%)	
$120,001 to $130,000	238 (11.2%)	78 (14.5%)	
More than $130,000	132 (6.2%)	50 (9.3%)	
**Incentive for education loan repayment influenced decision to accept position**			
No	1290 (59.6%)	224 (41%)	< .001
Yes	873 (40.4%)	322 (59%)	

## QUALITATIVE FINDINGS

Table 7 presents themes from open-ended comments regarding educational debt influencing choice or preference for primary care versus nonprimary care specialties.

- **PAs who accepted a position in primary care and said that debt influenced their choice (n = 201; 20.3%)**. Of the 201 respondents, 192 (95.5%) provided a comment elaborating on how educational debt affected their specialty choice. The most prevalent theme mentioned in 99 comments from this group of PAs was related to more opportunities in primary care for student loan repayment, forgiveness, or scholarship programs. Many indicated that they were National Health Service Corps (NHSC) scholarship recipients graduating with no debt but a requirement to work in primary care in underserved areas for a specified period of time. A few noted that some states offered educational debt relief programs, and a similar number indicated joining the military as a PA in primary care for assistance with paying off loans. Other less-prevalent themes included accepting a position in primary care to start paying off debt sooner rather than waiting for a position in other specialties, not wanting to complete a fellowship or residency, primary care being available in a preferred location, and graduating with low or no debt that allowed the PA to choose a preferred specialty without worrying about salary.
- **PAs who accepted a position in nonprimary care specialties and said that debt influenced their choice (n = 402; 11.9%)**. Of these respondents, 391 (97.3%) further described (in an open-ended question) how they were influenced by educational debt in their choice to accept a position in a nonprimary care specialty. The dominant theme, mentioned in 244 comments, was related to specialty practice offering higher pay than primary care, making it easier for PAs to pay off debt more quickly.
  The second most prevalent theme was that PAs sought specialty practice positions with the option of public service loan forgiveness, such as those offered in nonprofit hospitals. Some PAs said that specialty practice offers the possibility of extra shifts, more flexibility, better work-life balance, opportunity for fellowship or residency, availability of positions in preferred geographic locations, and more favorable family/cost of living considerations. The last theme was related to personal preference. Some PAs said they preferred primary care, but high educational debt and lower pay necessitated choosing positions in other specialties.
- **PAs who said that debt influenced their choice to seek a position in primary care (n = 94; 15.4%)**. Of the 94, almost all (93; 99.9%) provided a comment. The central theme among this group of PAs (42 comments) was related to paying off educational debt as a priority through loan repayment options or being an NHSC scholarship recipient, which requires primary care practice for a specified time period.
- **PAs who said that debt influenced their choice to seek a position in a nonprimary care specialty (n = 396; 21.2%)**. Lastly, 383 (96.7%) of PAs further described how educational debt influenced their choice to pursue a position in other specialties. The most common theme was that specialty practice offers higher income and financial security, enabling PAs to pay off educational debt more easily and quickly (347 comments). The second most prevalent theme was related to seeking nonprimary care positions with loan repayment options (43 comments). Another theme was related to the conflict between passion for specialty versus debt. Some PAs felt they would prefer to work in primary care but would seek other specialties because of the large amount of educational debt accrued and the need to make sufficient income. Others mentioned postponing primary care and working in higher-paying specialties until their educational debt was paid off. Finally, a smaller number of comments highlighted less favorable aspects of primary care positions, including long working hours, increased risk of burnout, and less flexibility.

**TABLE 7.** Themes from open-ended comments about educational debt influencing choice or preference for primary care vs. nonprimary care specialties

**PAs who accepted a position and indicated that debt influenced their choice**	
**Accepted a position in primary care (n = 201; 20.3%)**	**Accepted a position in nonprimary care specialties (n = 402; 11.9%)**
More opportunities in primary care for student loan repayment, forgiveness, or scholarship programs being available	Specialty practice offers higher pay than primary care, which allows for making monthly loan payments more easily and paying off debt more quickly
NHSC scholarship recipients graduating with no debt but with the requirement of working in primary care in underserved areas for a specified period of time	Sought specialty practice positions with the option of public service loan forgiveness, such as those offered in nonprofit hospitals
Particular states offered educational debt relief programs	Specialty practice offers the possibility of extra shifts
Joining the military as a PA in primary care for assistance with paying off loans	Specialty practice offers more flexibility and better work-life balance
Accepting a position in primary care to start paying off debt sooner rather than waiting for a position in other specialties	Opportunity for fellowship/residency
Not wanting to complete a fellowship/residency	Availability of positions in preferred geographic locations
Primary care being available in a preferred location	Family/cost of living considerations
Graduating with low or no debt allowed for choosing a preferred specialty without worrying about salary	Preferred primary care, but high educational debt and lower pay necessitated choosing positions in other specialties
**PAs seeking a position who indicated that debt influenced their preference**	
**Seeking a position in primary care (n = 94; 15.4%)**	**Seeking a position in nonprimary care specialty (n = 396; 21.2%)**
Paying off educational debt as a priority through loan repayment options or being an NHSC scholarship recipient, which requires primary care practice for a specified time period	Specialty practice offers higher income and financial security, enabling paying off educational debt more easily and quickly
	Seeking nonprimary care positions with loan repayment options
	Prefer to work in primary care but would seek other specialties due to the large amount of educational debt accrued and the need to make sufficient income
	Postponing primary care and working in higher-paying specialties until educational debt is paid off
	Less favorable aspects of primary care positions (for example, long working hours, increased risk of burnout, and less flexibility)

## DISCUSSION

Our study explored the complex interplay between educational debt, individual factors, and repayment strategies and their collective effect on specialty choices and preferences for PAs' first clinical positions. Three major findings emerged from our analyses. First, educational debt accrued was significantly associated with specialty choices or preferences: PAs with higher debt were more likely to opt for nonprimary care specialties. However, only 13.8% of PAs who accepted a position indicated that debt influenced their specialty choice; the proportion was slightly higher (20.2%) for PAs who had not yet accepted a position.

Second, PAs who indicated that educational debt influenced their specialty choice or preference had higher starting salaries or salary expectations, and were more likely to accept or pursue positions offering loan repayment. The themes from the detailed qualitative responses suggest that PAs rely on different strategies for repaying their loans, depending on whether they choose or prefer primary care or nonprimary care specialties.

Third, a concerning finding was that PAs who were older or from URiM groups had accrued higher educational debt than their counterparts.

Our study aligns with previous literature, confirming that higher educational debt often is associated with choosing nonprimary care specialties.^12^,^16^,^20^,^23^ For instance, Twombly and colleagues discovered that 28% of PAs who chose nonprimary care disciplines cited educational debt as a significant influence on their decision, compared with 9% of those who opted for primary care.^20^ However, in our study, only 13.8% and 20.2% reported that debt influenced their choice or preference, respectively. For both groups (accepted a position and still searching), educational debt accrued was positively associated with an increasing likelihood of agreeing that debt was influential on their choice or preference. Nonetheless, even among the PAs with the highest level of educational debt ($200,000 or more), 11.3% said debt had no effect on their specialty choice or preference. This finding suggests that although debt plays a role in specialty selection, other factors also are important and must be considered.^15^,^24^

Our second major finding is related to the interplay between choosing particular specialties and different strategies PAs adopt to repay their educational loans. PAs who said educational debt affected their choice or preference were more likely to have higher starting salaries or salary expectations, as well as to accept or pursue positions offering loan repayment. In our qualitative analyses, we discovered a prevailing theme among those who selected or favored nonprimary specialties: these roles offer higher salaries, which could expedite loan repayment; many felt that they had more debt than could be supported by income from primary care positions. Many also suggested that employment in nonprofit hospitals could lead to loan forgiveness over time, a benefit not available with primary care roles in private, office-based practices.

On the other hand, PAs who chose or preferred primary care often cited the availability of NHSC scholarship programs, which can significantly reduce or even eliminate educational debt. PAs have a longstanding tradition of participating in such programs.^25^ Many noted opportunities for working in federally qualified health centers, rural health clinics, and state and local public health clinics that offer federal Public Service Loan Forgiveness programs. A common sentiment, regardless of specialty choice or preference, was the flexibility to switch disciplines in the future. Some PAs intended to complete their NHSC scholarship requirement and switch from primary care to other specialties. Similarly, some who chose nonprimary care specialties intend to pay off their loans quicker because of the higher income and then practice in primary care. A similar theme emerged in a study conducted by the Robert Graham Center from seven focus group interviews with second-year PA students who expected to practice in a variety of specialties throughout their careers.^19^ This flexibility, a unique characteristic of the PA profession, lets PAs leverage opportunities in both primary care and other specialties to repay their loans as quickly as possible.

A concerning finding in our analysis was that PAs from URiM groups reported higher debt levels. Recent studies reported similar findings with pediatric interns, physical therapy students, postgraduate medical residents, and medical school graduates.^2^,^10^,^26^,^27^ For instance, in 2019, 61% of Asian and 75% of White medical school graduates had educational debt. However, this figure was highest (91%) for Black graduates.^2^ Furthermore, among those with debt, the median amount was also highest for Black medical school graduates ($230,000) compared with Asian ($180,000) and White ($200,000).^2^ Evidence indicates that clinicians from URiM backgrounds are more likely to practice in primary care and provide care to patients in underserved areas.^28^,^29^ Muma and colleagues, in a sample of 10,500 PAs, found that PAs from URiM groups, compared with their counterparts, were more likely to care for underserved patients and to work in primary care.^28^ In our study, among PAs who accepted a position, PAs who identified as Black and Hispanic/Latinx were more likely to choose primary care. Also, PAs who identified as Black were more likely to indicate that debt influenced their specialty choice. However, more research is needed to better understand how educational debt influences specialty choices for PAs from URiM groups.

Taken together, our findings on educational debt and specialty choice for recently certified PAs entering the healthcare workforce provide valuable insights to policymakers, PA programs, and higher education institutions to help increase the number of PAs working in primary care settings and develop financial incentives or compensation models to make certain specialties with clinician shortages more attractive. For instance, existing loan forgiveness and repayment could be expanded to target PAs from URiM groups, because we found higher debt among those PAs. Federal loan forgiveness and repayment programs generally support primary care medical specialties, and those who choose to participate in these programs are more likely to work in medically underserved areas and healthcare professional shortage areas.^30^ However, more studies are needed to elucidate how loan forgiveness and repayment programs affect PA practice patterns. Furthermore, scholarship options could be included in conversations with undergraduate students interested in healthcare in order to attract a diverse group of applicants. The PA community should explore scholarship options for pre-PA students interested in this field, particularly for students interested in primary care specialties and students from URiM groups.^31^ Nonetheless, we cannot assume that medical specialty selection is determined by educational debt alone, and other factors may be influential. Further research should investigate how specialty choice for newly certified PAs is influenced by other potential confounders such as socioeconomic components, personal interests, opportunities, skills, mentors/advisors, and life experiences.

## LIMITATIONS

Our study is a cross-sectional study, which inherently precludes establishing causal relationships. Secondly, the data are based on self-reported responses, which could introduce biases related to social desirability and recall. The third limitation pertains to the response rate. Of the 11,762 PAs who completed their initial certification in 2023, 7,494 (63.7%) responded to the question about their acceptance of a PA position. The Recently Certified Module, which collects these responses, only is available for 1 year after initial certification. This limited timeframe may have deterred some PAs from participating. Lastly, our study did not assess several potentially influential factors. These include attributes of PA schools (such as clinical experiences), availability of positions, geographic preferences, the effect of mentoring and role modeling, family influences, and marital status. These factors could provide a more comprehensive understanding of PAs' specialty choices and preferences and should be considered for future studies.

## CONCLUSIONS

PAs with higher educational debt were more likely to choose nonprimary care specialties; however, only a small percentage acknowledged that debt influenced their specialty choice. PAs who stated that debt influenced their specialty choice had higher starting salaries or salary expectations and were more likely to accept or prefer positions offering loan repayment. The study also revealed that PAs use different loan repayment strategies depending on whether they choose primary care or nonprimary care specialties. A concerning finding was that PAs from URiM groups had higher educational debt than their counterparts.
